# A Randomized Trial Directly Comparing Ventral Capsule and Anteromedial Subthalamic Nucleus Stimulation in Obsessive-Compulsive Disorder: Clinical and Imaging Evidence for Dissociable Effects

**DOI:** 10.1016/j.biopsych.2019.01.017

**Published:** 2019-05-01

**Authors:** Himanshu Tyagi, Annemieke M. Apergis-Schoute, Harith Akram, Tom Foltynie, Patricia Limousin, Lynne M. Drummond, Naomi A. Fineberg, Keith Matthews, Marjan Jahanshahi, Trevor W. Robbins, Barbara J. Sahakian, Ludvic Zrinzo, Marwan Hariz, Eileen M. Joyce

**Affiliations:** a Department of Clinical and Movement Neurosciences, University College London Queen Square Institute of Neurology, London, United Kingdom; b The National Hospital for Neurology and Neurosurgery, London, United Kingdom; c Highly Specialised Service for OCD and BDD (England), SW London and St George's NHS Trust, London, United Kingdom; d Department of Psychology, Behavioural and Clinical Neuroscience Institute, University of Cambridge, Cambridge, United Kingdom; e Department of Psychiatry, Behavioural and Clinical Neuroscience Institute, University of Cambridge, Cambridge, United Kingdom; f Highly Specialised Service for OCD and BDD (England), Hertfordshire Partnership University NHS Foundation Trust, Welwyn Garden City, United Kingdom; g Centre for Clinical & Health Research Services, School of Life and Medical Sciences, University of Hertfordshire, Hatfield, United Kingdom; h Division of Neuroscience, School of Medicine, University of Dundee, Dundee, United Kingdom; i Advanced Interventions Service, NHS Tayside, Ninewells Hospital and Medical School, Dundee, United Kingdom

**Keywords:** Anteromedial subthalamic nucleus, DBS, Deep brain stimulation, Obsessive-compulsive disorder, OCD, Ventral internal capsule

## Abstract

**Background:**

Deep brain stimulation (DBS) is an emerging treatment for severe obsessive-compulsive disorder (OCD). We compared the efficacy of ventral capsule/ventral striatal (VC/VS) and anteromedial subthalamic nucleus (amSTN) DBS in the same patients and tested for mechanistic differences on mood and cognitive flexibility and associated neural circuitry. The possible synergistic benefit of DBS at both sites and cognitive behavioral therapy was explored.

**Methods:**

Six patients with treatment-refractory OCD (5 men; Yale-Brown Obsessive Compulsive Scale score >32) entered double-blind counterbalanced phases of 12-week amSTN or VC/VS DBS, followed by 12-week open phases when amSTN and VC/VS were stimulated together, in which optimal stimulation parameters were achieved and adjunctive inpatient cognitive behavioral therapy was delivered. OCD and mood were assessed with standardized scales and cognitive flexibility with the Cambridge Neuropsychological Test Automated Battery Intra-Extra Dimensional Set-Shift task. Diffusion-weighted and intraoperative magnetic resonance imaging scans were performed for tractography from optimally activated electrode contacts.

**Results:**

DBS at each site significantly and equivalently reduced OCD symptoms with little additional gain following combined stimulation. amSTN but not VC/VS DBS significantly improved cognitive flexibility, whereas VC/VS DBS had a greater effect on mood. The VC/VS effective site was within the VC. VC DBS connected primarily to the medial orbitofrontal cortex, and amSTN DBS to the lateral orbitofrontal cortex, dorsal anterior cingulate cortex, and dorsolateral prefrontal cortex. No further improvement followed cognitive behavioral therapy, reflecting a floor effect of DBS on OCD.

**Conclusions:**

Both the VC/VS and amSTN are effective targets for severe treatment-refractory OCD. Differential improvements in mood and cognitive flexibility and their associated connectivity suggest that DBS at these sites modulates distinct brain networks.

SEE COMMENTARY ON PAGE 706

Obsessive-compulsive disorder (OCD) has a lifetime prevalence of 1% to 2%. Effective treatments include cognitive behavioral therapy (CBT) and serotonin reuptake inhibitor medication (1), but there remains a severely impaired, treatment-refractory subgroup (2) for whom anterior cingulotomy or capsulotomy is an option (3).

Deep brain stimulation (DBS) for OCD was introduced as an alternative to neurosurgical ablation because it is reversible and adjustable (4). Four studies have included a randomized controlled trial of DBS at the site of the anterior capsulotomy (ventral capsule/ventral striatum [VC/VS]). Earlier, small studies found that relatively few patients responded to VC/VS DBS 5, 6, but more recent, larger studies found level 1 evidence in favor of DBS 7, 8. Neurosurgical targeting in these studies changed over time (9), and there were also differences in the choice of DBS electrodes, all of which had four contacts at their tip but variable distances between them. It is likely that such factors led to discrepancies in the location and/or spread of stimulation and may explain the inconsistent response rates. For example, within the area of the VC/VS, it is not clear whether stimulation of capsular white matter and/or nearby gray matter provides the optimal response 7, 8.

The anteromedial subthlamic nucleus (amSTN) is an alternative DBS target for OCD following a randomized controlled trial by Mallet *et al.* (10), who found significant symptomatic reduction. Which of the two sites is better for ameliorating severe OCD has yet to be established. In addition, preliminary evidence suggests that the mechanisms of action at the two sites may differ. VC/VS DBS studies have reported a pronounced improvement in mood 5, 7, 8 not evident following the amSTN study (10), leading Denys *et al.* (7) to suggest that different neural circuits may mediate OCD, with one stimulated by VC/VS DBS primarily improving mood and another affected by amSTN DBS primarily modulating compulsive behavior.

In this study, using a randomized, double-blind, counterbalanced design, we investigated the effects of VC/VS and amSTN DBS in the same patients to 1) establish whether one site is more efficacious than the other in improving OCD, 2) determine the precise neuroanatomical locations for optimal effects by calculating volumes of tissue activation (VTAs) at each site, and 3) test the hypothesis of Denys *et al.* (7) by first employing ratings of mood and a test of cognitive flexibility previously shown to measure an endophenotype of OCD (11) and then investigating the neural circuitry associated with the DBS at each site with magnetic resonance imaging (MRI) tractography.

The potential of synergistic benefit of DBS at both sites and adjunctive CBT was additionally explored in open phases when both DBS sites were stimulated.

## Methods and Materials

### Participants

Six patients were recruited from OCD specialized services. Eligibility required ICD-10 OCD, age >20 years, illness duration >10 years, unremitting symptoms for 2 years, Yale-Brown Obsessive Compulsive Scale (Y-BOCS) score ≥32, and DSM-IV General Assessment of Function Scale score ≤50. Patients were ineligible if they were pregnant or had ICD-10 substance misuse, organic brain syndrome, adult personality disorder (except obsessive-compulsive type), pervasive developmental disorder, schizophrenia or bipolar disorder, or contraindications to neurosurgery. Treatment resistance was defined as no sustained benefit from 1) at least two serotonin reuptake inhibitors for a minimum of 12 weeks at optimal doses; 2) augmentation of serotonin reuptake inhibitor treatment with antipsychotics or extension of the serotonin reuptake inhibitor dose beyond recommended limits; and 3) two trials of CBT, one as inpatient, lasting 10 hours minimum. All patients provided written informed consent. The clinical trial was registered with the UK Clinical Research Network (No. 13158) and the ISRCTN registry (No. 18430630) and received ethical approval (clinical study: National Health Service Health Research Authority No. 12/LO/1087; MRI: National Health Service West London Research Ethics Committee No. 10/H0706/68).

### Study Design

Eligible participants were offered stereotactic ablation or entry to the DBS trial. Prescribed medications were kept constant unless clinically indicated. Before surgery, all patients underwent 3T diffusion-weighted MRI for tractography analysis (Magnetom Tim Trio; Siemens, Berlin, Germany). Following surgery, stimulation remained off for at least 4 weeks. At the beginning of each phase, participants were admitted to a neuropsychiatry ward for 2 weeks for DBS adjustment. Participants remained on optimized settings for 12 weeks with a contingency for readjustment if clinically indicated (Supplemental Figure S1).

The initial two phases were double blind, randomized, and counterbalanced; each phase lasted 12 weeks. Participants received stimulation of either the amSTN (*n =* 3) or the VC/VS (*n =* 3) followed by the alternate condition. A 12-week open phase followed, during which electrodes at both sites were active (combined stimulation [COMB] phase). There were two additional 12-week open phases when optimized stimulation settings were delivered using data from previous phases (OPT phase), followed by the participants’ receiving CBT/exposure and response prevention in an inpatient unit (OPT plus adjunctive CBT phase) while continuing with the OPT DBS settings. Clinical and cognitive assessments were performed before surgery (baseline) and after each phase.

### Neurosurgery

Surgery was performed under general anesthesia. Patients underwent stereotactic 1.5T MRI (Magnetom Avanto; Siemens) for planning of amSTN and VC/VS coordinates and trajectories (12). Through 14-mm frontal bilateral burr holes, 1.5-mm-diameter radiofrequency electrodes were introduced to each target under dynamic impedance monitoring. These were replaced with DBS leads through the same trajectory to the target. Separate corticotomies, within the same burr hole, were used to implant the two DBS leads. Quadripolar DBS leads were used with 0.5-mm separation between contacts for amSTN leads (3389; Medtronic, Minneapolis, MN) and 1.5-mm separation between contacts for VC/VS leads (3387; Medtronic). The VC/VS lead was planned to locate one contact within the nucleus accumbens core, one contact within its shell, and the upper two contacts in the most ventral aspect of the anterior limb of the internal capsule. An immediate stereotactic MRI verified targeting accuracy (13). Two neurostimulators (Activa PC; Medtronic) were placed subcutaneously below the collarbone, one on each side, and each was connected to bilateral leads from one of the electrodes via subcutaneous cables.

### Randomization and Blinding

Computer-generated pairwise randomization was used so that equal numbers were recruited to receive amSTN or VC/VS stimulation first, in a counterbalanced order. Two unblinded clinicians (TF, PL) held the randomization list and adjusted DBS parameters. All other team members, ward staff, and participants were blinded to allocation.

### Stimulus Adjustments

Optimal DBS parameters were derived in an iterative fashion over 2 weeks. Initially, each contact was screened with voltages up to 4 V (amSTN) or 8 V (VC/VS) using monopolar stimulation (pulse width 60 μs and frequency 130 Hz). Immediate clinical effects from stimulation delivered through each contact in turn and the threshold associated with positive and negative effects were noted. Anticipated adverse effects of stimulation included hypomania and anxiety 9, 10 and were documented with a visual analog scale. Stimulation parameters were refined daily according to patient feedback, visual analog scale, and clinical assessment, including the use of stimulation through multiple monopolar electrode contacts per lead or using a bipolar configuration.

### Clinical and Cognitive Assessments

The Y-BOCS was the primary outcome measure to test VC/VS and amSTN DBS effects on OCD symptoms. To test the mechanistic hypothesis, secondary measures were the Montgomery–Åsberg Depression Rating Scale (MADRS) to evaluate mood and the Cambridge Neuropsychological Test Automated Battery Intra-Extra Dimensional Set-Shift (IED) task to evaluate cognitive flexibility. In the IED task, participants progress through nine stages assessing the ability to learn and reverse rules governing correct responses using computer feedback (14). In stages 1 to 7, responses to a specific stimulus dimension are correct. The ability to shift attention away from the previously correct stimulus dimension to a different dimension (i.e., cognitive flexibility) is tested in extradimensional set-shifting task stage 8 (EDS).

### Volumes of Tissue Activation

SureTune, Version 2 (Medtronic), a DBS therapy planning platform, was used to model activation volumes around individual contacts. This applies neuron models coupled to finite element simulations to generate DBS therapy activation volumes (15). The preprocessed postoperative magnetization prepared rapid acquisition gradient echo scans were manually aligned with the preimplantation magnetization prepared rapid acquisition gradient echo scans (see Supplement). Automatic coregistration was carried out with a restricted volume of fusion cantered on the mesencephalon, diencephalon, and VS to minimize registration error resulting from brain shift incurred during surgery despite minimal brain shift with our surgical technique (16). Registration accuracy was inspected and the process iterated if necessary. All volumes were realigned with a plane parallel to the anterior commissure-posterior commissure line.

The postimplantation magnetization prepared rapid acquisition gradient-echo scan was used to fit the DBS lead model within the MRI artifact produced by the leads. For each patient, DBS parameters during the 12-week phase when each set of electrodes was active and optimized were used to generate activation volumes around active DBS contacts in the amSTN and VC/VS with corresponding voltages. Binary image files of activation volumes with corresponding transformation matrices were exported and processed in MATLAB, version R2016A (The MathWorks, Inc., Natick, MA) using an in-house software to generate Neuroimaging Informatics Technology Initiative volumes for further analysis.

Volumes corresponding to the active electrodes for each target were merged using Fslmerge (FSL v5.0) into a 4-dimensional data file for each hemisphere. Fslmaths (FSL v5.0) was then used to generate group average volumes of tissue activation.

### Connectivity Analysis

Preprocessed data (see Supplement) were fed into BedpostX (FSL v5.0) to estimate fiber orientations. Up to three crossing fibers were estimated in each brain voxel using model 2 and graphics processing unit parallelization 17, 18. Probtrackx was used on these estimates to obtain global connectivity (i.e., the probability of the existence of a path through the diffusion field between any two distant points, a surrogate measure of anatomical connectivity) (19). Probabilistic tractography was generated in ProbtrackX2 graphics processing unit version (FSL v5.0) (number of samples = 5000, curvature threshold = 0.2, step length = 0.5 mm, subsidiary fiber volume fraction threshold = 0.01) (19). The process repetitively samples from the distributions of voxelwise principal diffusion directions generated in BedpostX, each time computing a streamline through these local samples to generate a probabilistic streamline or a sample from the distribution on the location of the true streamline, building up a spatial connectivity distribution. Streamlines truly represent paths of minimal hindrance to diffusion of water in the brain, but they are reasonable indirect estimates of long-range white matter connections (20).

Probabilistic tractography streamlines were generated for each patient in native space. Individual patient DBS VTAs were used as seed masks resulting in four tracts per patient from the amSTN and the VC/VS bilaterally. The corpus callosum was used as an exclusion mask. Cerebrospinal fluid termination masks were used to exclude false positive streamlines. Using the obtained transformations to and from standard space, resulting streamlines were transformed to Montreal Neurological Institute space, and group averages were generated. *The Human Central Nervous System: A Synopsis and Atlas* (21) was used to corroborate the relevant anatomical structures.

### Statistical Analysis

Friedman’s test was used to test for DBS effects during the double-blind crossover phases comparing baseline, amSTN, and VC/VS. Significance levels were adjusted for multiple comparisons using the false discovery rate (FDR) method of Benjamini and Hochberg (22). Post hoc pairwise Conover tests for significant effects were used (23), with FDR corrections also applied. For each variable, the effect of time, stimulation at both sites, and adjunctive CBT was assessed with Friedman's test across all six time points independent of stimulation type. To test the effect of DBS of amSTN and VC/VS together, Wilcoxon’s tests were used to compare phases when one site was stimulated (time 2 + time 3) and when both sites were stimulated (time 4 + time 5) and the effect of CBT, by comparing time 5 and time 6. FDR corrections of significance values were applied throughout. To substantiate our effects, we repeated these analyses using more powerful parametric statistics, again correcting for multiple comparisons (see Supplement).

## Results

Six participants were recruited from NHS England Specialised OCD Services by HT, NF, LMD, and KM. Five were men; the age range was 38 to 62 years and the duration of illness was 20 to 30 years (Table 1). The study took place between January 10, 2013, and October 25, 2016. During the trial, patient 2 required further DBS adjustment during the amSTN, VC/VS, and COMB phases and required additional medication for mood instability. Patient 6 required DBS adjustment during the OPT phase because of worsening of OCD. Patient 1’s OCD symptoms improved during VC/VS DBS but worsened when switched to amSTN. Stimulation adjustment over 2 weeks made no improvement. Because of patient distress, stimulation reverted to the VC/VS site. The scores entered for the amSTN stage for this participant were at the point of switching from amSTN back to VC/VS, akin to last entry carried forward. This participant continued with subsequent trial phases per protocol.

**Table 1:** Patient Details

Patient No./Sex	Age at Onset, Years	Age at Surgery, Years	Illness Duration, Years	Disability at Study Entry	Medication
1/Female	16	38	22	Living in inpatient unit; failed supportive accommodation	Escitalopram 20 mg; risperidone 0.5 mg; trazodone 150 mg; clomipramine 200 mg
2/Male	16	38	22	Extreme avoidance; impaired social function	Tramadol 200 mg; memantine 35 mg; pregabalin 300 mg; citalopram 120 mg; quetiapine 25 mg; clonazepam 0.75 mg
3/Male	32	62	30	Largely housebound; requiring help with ADLs	Aspirin 75 mg; omeprazole 20 mg; sitagliptin 100 mg; nitrazepam 10 mg as needed
4/Male	17	37	20	Largely housebound; severely impaired in ADLs	Escitalopram 40 mg; aripiprazole 10 mg
5/Male	32	55	23	Largely housebound; unable to live independently	Aripiprazole 20 mg; chlorpromazine 400 mg; pregabalin 300 mg; propranolol 40 mg; sertraline 400 mg; zopiclone 7.5 mg
6/Male	15	43	28	Extreme avoidance; impaired social function	Pregabalin 600 mg; aripiprazole 20 mg; sertraline 200 mg

ADLs, activities of daily living.

### Comparison of VC/VS and amSTN DBS

**Figure 1:**
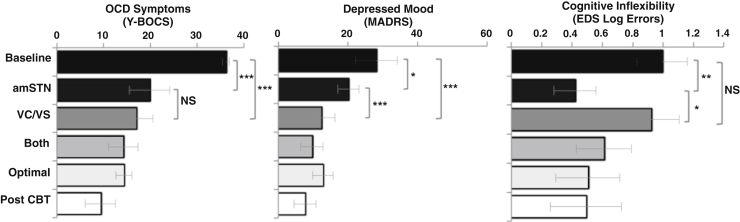
Mean Yale-Brown Obsessive Compulsive Scale (Y-BOCS) score, Montgomery–Åsberg Depression Rating Scale (MADRS) score, and extra-dimensional set-shifting (EDS) log errors at baseline and following deep brain stimulation phases: anteromedial subthalamic nucleus (amSTN), ventral capsule/ventral striatum (VC/VS); combined amSTN and VC/VS DBS (Both); optimal combined settings (Optimal); optimal combined settings plus cognitive behavioral therapy (Post CBT). The amSTN and VC/VS phases followed a randomized counterbalanced design. Both the Optimal and Post-CBT phases were open and sequential. Y-BOCS scores range from 1 to 40: 0–7 = subclinical; 8–15 = mild; 16–23 = moderate; 24–31 = severe; 32–40 = extreme. MADRS scores range from 1 to 60: 0–6 = normal; 7–19 = mild; 20–34 = moderate; 35–60 = severe. *p < .05; **p < .01; ***p ≤ .001. NS, not significant; OCD, obsessive-compulsive disorder.

All participants completed the IED task, and the only significant DBS effect was an improvement in EDS errors. There were significant improvements in Y-BOCS score (Table 2), MADRS score, and EDS errors following DBS (Figure 1). All Friedman tests were significant, controlling for FDR: Y-BOCS score (χ^2^_2_ = 9.0, *p =* .017), MADRS score (χ^2^_2_ = 10.33; *p =* .017), EDS errors (χ^2^_2_ = 7.00, *p =* .03). Y-BOCS scores significantly improved following both amSTN and VC/VS DBS (baseline vs. amSTN: *p <* .001; baseline vs. VC/VS: *p <* .001; amSTN vs. VC/VS: *p* = 1.00). Changes in MADRS scores were significantly different from baseline for both amSTN and VC/VS DBS. The magnitude of the amSTN effect was significantly greater than VC/VS (baseline vs. amSTN: *p =* .023; baseline vs. VC/VS: *p <* .001; amSTN vs. VC/VS: *p =* .001). Changes in EDS errors were significant for amSTN but not VC/VS DBS (baseline vs. amSTN: *p =* .003; baseline vs. VC/VS: *p =* .157; amSTN vs. VC/VS: *p =* .018). Parametric analyses substantiated these effects other than the post hoc comparison of baseline and amSTN DBS on the MADRS, which was not significant (see Supplement).

**Table 2:** Y-BOCS Scores at Baseline and During Five Stimulation Phases

Patient	Baseline	amSTN	VC/VS	COMB	OPT	AdCBT
1	38	32 (16)^a^	22 (42)	17 (55)	18 (53)	13 (66)
2	34	26 (23)	29 (15)	23 (32)	20 (41)	21 (38)
3	37	17 (55)	18 (51)	12 (68)	10 (73)	2 (95)
4	38	20 (47)	13 (66)	16 (58)	10 (74)	7 (82)
5	34	23 (32)	17 (50)	17 (50)	15 (56)	13 (62)
6	36	1 (97)	3 (92)	0 (100)	13 (64)	0 (100)
Mean ± SEM	36.17 ± 0.75	19.83 ± 4.32	17.00 ± 3.57	14.17 ± 3.18	14.33 ± 1.69	9.33 ± 3.21

Values in parentheses indicate % reduction from baseline. amSTN deep brain stimulation was the initial condition for patients 4, 5, and 6.

AdCBT, optimal combined settings plus adjunctive cognitive behavioral therapy; amSTN, anteromedial subthalamic nucleus; COMB, combined phase; OPT, optimal combined settings; VC/VS, ventral capsule/ventral striatum; Y-BOCS, Yale-Brown Obsessive Compulsive Scale.

a This score is last entry carried forward. Y-BOCS scores range from 1 to 40 and are categorized according to severity as follows: 0–7 = subclinical; 8–15 = mild; 16–23 = moderate; 24–31 = severe; 32–40 = extreme.

Previous studies have defined a DBS response as ≥35% reduction in baseline Y-BOCS 5, 6, 7, 8. The proportion of patients achieving responder status at the end of each phase was the following: amSTN phase, 3 of 6; VC/VS phase, 5 of 6; COMB phase, 5 of 6; OPT phase, 6 of 6; OPT plus adjunctive CBT phase, 6 of 6.

### Effects of Time, Combined DBS, and Adjunctive CBT

Following FDR corrections across all three Friedman’s tests, there was a significant improvement in Y-BOCS and MADRS scores over time, and EDS errors did not change: Y-BOCS score (χ^2^_5_ = 24.11, *p <* .001), MADRS score (χ^2^_5_ = 16.49, *p =* .006), EDS errors (χ^2^_5_ = 6.59, *p =* .253). Following FDR corrections of post hoc comparisons, there were no statistically significant changes when combined DBS of both sites was compared with single-site stimulation (Y-BOCS: *p =* .116; MADRS: *p =* .146) or when DBS at both sites was compared with adjunctive CBT (Y-BOCS: *p =* .092; MADRS *p =* .223). Parametric analyses substantiated these findings (see Supplement).

### Amplitude of Active Contacts VTAs

**Figure 2:**
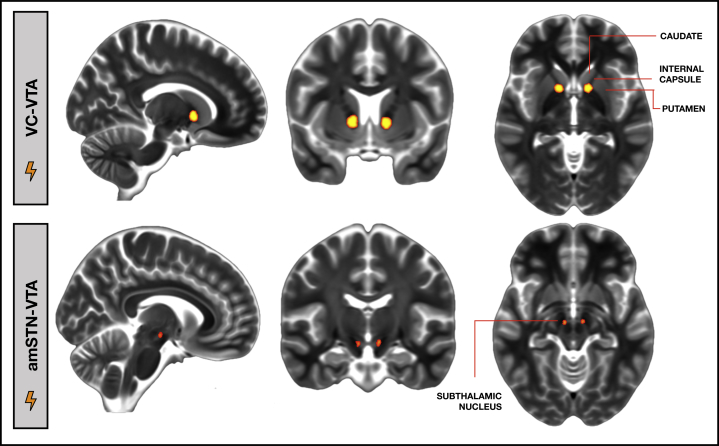
Average deep brain stimulation volume of tissue activation (VTA) in the ventral capsule (VC-VTA) and anteromedial subthalamic nucleus (amSTN-VTA).

Postoperative stereotactic MRI verified that amSTN electrodes had at least two contacts within the amSTN and that VC/VS electrodes had two or three contacts within the VC and one or two contacts in the nucleus accumbens. For VC/VS, the most dorsal contacts produced the optimal clinical response for all patients, and the mean stimulation amplitude was 5.85 ± 1.2 V (Supplemental Tables S1 and S2). For the amSTN, the deepest contacts were most effective for all patients, and the mean stimulation amplitude was 1.56 ± 0.82 V (Supplemental Tables S1 and S2). The average VC/VS VTA was centered on the white matter in the ventral portion of the anterior limb of the internal capsule and encroached slightly on adjacent portions of the nucleus accumbens, head of caudate, globus pallidus, and putamen (Figure 2). The average amSTN VTA was centered on the anterior-inferior medial border of the STN spreading into the ventral tegmental area (Figure 2). Exploratory multiple regressions showed that changes in Y-BOCS score, MADRS score, or EDS errors did not predict STN and VC volumes of tissue activation following STN and VC DBS, respectively (range of *B* values: −0.750 to 0.625; range of *t* values: −1.988 to 1.254).

### Tractography

**Figure 3:**
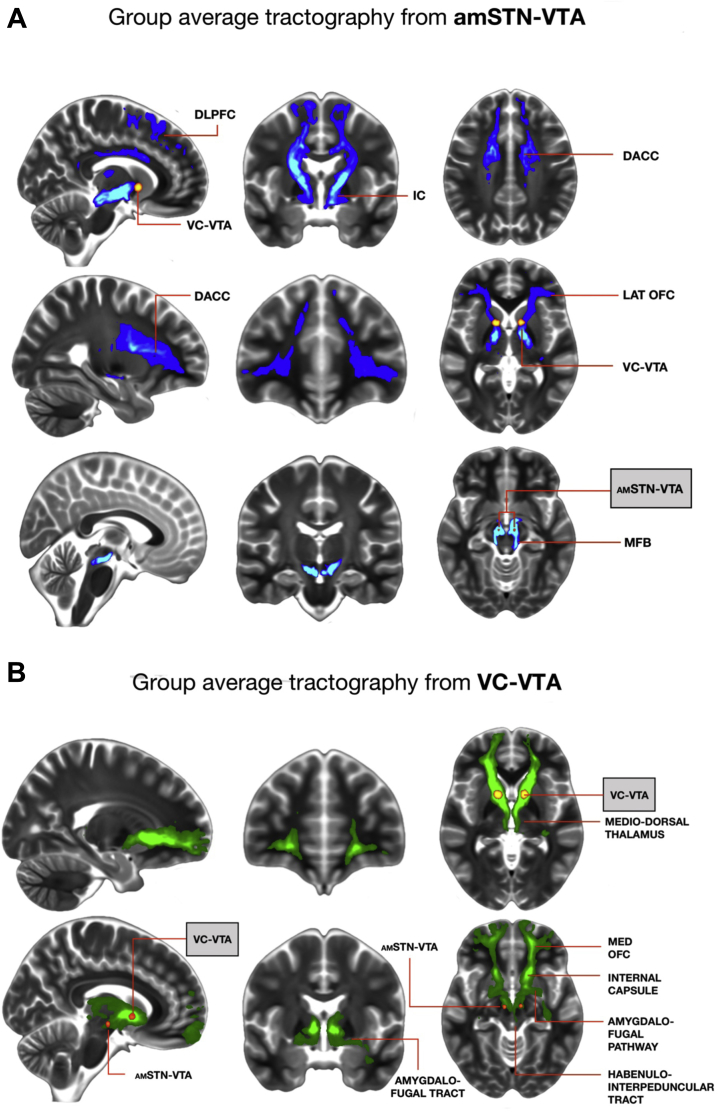
**(A)** Group average streamlines from anteromedial subthalamic nucleus volumes of tissue activation (amSTN-VTA). **(B)** Group average streamlines from ventral capsule volumes of tissue activation (VC-VTA). DACC, dorsal anterior cingulate cortex; DLPFC, dorsolateral prefrontal cortex; IC, internal capsule; LAT, lateral; MED, medial; MFB, medial forebrain bundle; OFC, orbitofrontal cortex.

The average streamlines generated from individual amSTN VTAs were connected to the lateral orbitofrontal cortex (OFC), dorsal anterior cingulate cortex (DACC), dorsolateral prefrontal cortex (DLPFC), and medial forebrain bundle. The average streamlines generated from individual VC VTAs were connected to the medial OFC, the mediodorsal thalamus, the amygdala via the amygdalofugal pathway, the hypothalamus, and the habenula via the habenulointerpeduncular tract (Figure 3A, B). Individual patient streamlines are shown in Supplemental Figure S2.

### Adverse Events

There were no adverse events associated with surgery. The most common adverse event during the DBS trial was hypomania (elevated mood or irritability, racing thoughts, disinhibition), occurring within hours after stimulation adjustment and remedied by further adjustment. This was witnessed twice each during amSTN and VC/VS DBS and three times during the COMB phase. One patient reported hypomania-like symptoms during the OPT phase on a more sustained basis (racing thoughts and urges to steal) and required frequent admissions for adjustment of DBS for unstable mood. The VC/VS battery became depleted and was replaced once in 3 patients and twice in 1 patient during open phases of the study, without surgical complications (Supplemental Table S3).

## Discussion

This is the first study to compare, in the same patients, DBS of two brain sites for severe OCD. In a within-subjects, randomized, double-blind, counterbalanced comparison of amSTN and VC/VS stimulation in 6 patients, we were able to address important questions regarding the efficacy and mechanism of DBS at each site. All patients had been ill for at least 20 years and failed to respond to high doses of medication plus intensive CBT. DBS significantly reduced OCD symptoms during stimulation of the VC/VS and amSTN, and the magnitude of the reduction at each site, measured with the Y-BOCS, did not differ. In contrast, there were different effects of DBS on mood and cognition. DBS of the amSTN, but not the VC/VS, significantly improved cognitive flexibility (EDS errors). DBS of the VC/VS elicited a striking improvement in mood, measured by the MADRS, which was to a greater degree than amSTN DBS. Tractography from optimally activated electrode contacts at each site suggests that these dissociated effects reflected DBS modulation of distinct brain networks.

Many studies suggest that orbitofrontal (OFC) cortico-striato-thalamic circuitry is dysfunctional in OCD. It is now recognized that the medial and lateral aspects occupy separate trajectories within the OFC cortico-striato-thalamic circuitry and are functionally different (24). Abnormal functional connectivity between lateral OFC (Brodmann areas 10, 11, 47) and caudate nucleus is associated with EDS errors in OCD (25). Compatible with this, amSTN DBS improves glucose metabolism in OFC (Brodmann areas 10, 11) in association with better Y-BOCS scores (26). The EDS tests the ability to adapt to changing circumstances by shifting the focus of attention and acquiring new responses (i.e., cognitive flexibility); this ability is a putative endophenotype of OCD, being also impaired in first-degree unaffected relatives (11). These findings are compatible with the observations of this study that the amSTN DBS site was associated with improved EDS performance and that tractography streamlines from activated contacts connected to the lateral OFC.

Connectivity analysis also showed streamlines linking the DLPFC and DACC with the amSTN. In addition to the OFC, the function of these two cortical areas has been linked to the severity of OCD symptoms measured by the Y-BOCS. For example, in a functional MRI study, reduced functional connectivity between the DLPFC and putamen was associated with both impaired goal-directed planning and OCD symptom severity measured by the Y-BOCS (25). In addition, ablation of the DACC, performed during cingulotomy, is an established neurosurgical procedure shown to alleviate symptoms in patients with medically refractory OCD (27).

In nonhuman primates, the amSTN receives uninterrupted projections from the OFC, DLPFC, and DACC, considered to form the limbic and cognitive components of the hyperdirect pathway (28). The STN is integral to basal ganglia circuitry, and input from the hyperdirect pathway provides a mechanism whereby cortical influences can rapidly suppress the manifestation of behaviors that are already being programmed (29). Taking all findings together, amSTN DBS may regulate aberrant information processing in the hyperdirect pathway from the lateral OFC, DLPFC, and DACC (28) and enable patients to interrupt their compulsive cycle of repetitive acts and thoughts through improving cognitive flexibility and goal-directed planning.

For all patients, the VC/VS dorsal-most electrode contacts were the most effective, suggesting that stimulation of the ventral anterior limb of the internal capsule (VC), rather than the VS/nucleus accumbens, mediated the clinical effect of improved OCD and mood. This was confirmed by the volume of tissue activation, which was centered on the VC with only minor encroachment on the surrounding gray matter. Other studies support this conclusion 8, 30.

Nonhuman primate tracing studies, shown to substantiate human tractography (31), suggest that VC DBS captures fibers from the medial OFC to the thalamus and more posterior areas 32, 33. This is in keeping with the tractography finding here of a streamline extending anteriorly to the medial OFC and posteriorly to the dorsomedial thalamus, as well as streamlines involving the amygdalofugal pathway and habenulointerpeduncular tract. The medial OFC is hyperactive during the early processing of threat-related stimuli in OCD (34). The amygdalofugal pathway is an output tract from the basolateral nucleus of the amygdala to the mediodorsal thalamus and OFC. A recent functional MRI study of OCD found specific, abnormal connectivity between the basolateral amygdala and medial OFC in OCD, which was predictive of successful treatment with CBT (35). The habenulointerpeduncular tract has been implicated in the development of depression via inhibition of brainstem serotonergic raphe nuclei (36). In OCD, selective serotonin reuptake inhibitors increase serotonin neurotransmission and are the mainstay of pharmacological treatment because they improve both mood and core OCD symptoms (37). Thus, modulation by VC DBS of circuitry involving the medial OFC, the associated amygdalofugal pathway, and the habenulointerpeduncular tract may mediate the striking improvement in mood as well as OCD symptoms.

We anticipated that a positive response to DBS would enable patients to utilize CBT and boost the OCD effect further (38), but this was not found. However, inspection of individual Y-BOCS changes before CBT showed that 4 of 6 patients were already at a mild level of OCD symptom severity, thus making it difficult for further measurable gains to be made during CBT.

Adverse effects were mainly stimulation-induced hypomania, also reported in other studies. These were not more common during stimulation of either site or combined stimulation.

There are several limitations to the study, the main one being the small sample size. However, when comparing the efficacy of the two DBS sites, patients served as their own controls in an innovative design, and the conclusions were robust to adjustment for multiple comparisons and parametric and nonparametric analyses. Nevertheless, it would be important to test our hypothesis in a larger group of patients when the mechanistic actions of STN and VC DBS on recovery from OCD can be evaluated in more detail. Another limitation is the possible confounding effect of combined stimulation and CBT with time. Future studies could compare the effect of additional CBT at an earlier stage.

In summary, DBS of the VC and amSTN significantly alleviated OCD symptoms, and the magnitude of effect did not differ between these sites, suggesting that both targets are equally efficacious. The finding that amSTN but not VC DBS improved cognitive flexibility and that the effect of DBS on mood was significantly greater for VC DBS implicates the involvement of different neural circuitries associated with distinct symptoms in OCD. Tractography findings revealed that VC and amSTN DBS modulate distinct brain networks implicated in OCD and are compatible with these clinical and cognitive observations.
