# Supplementary material for: A Randomized Trial Directly Comparing Ventral Capsule and Anteromedial Subthalamic Nucleus Stimulation in Obsessive-Compulsive Disorder: Clinical and Imaging Evidence for Dissociable Effects
Source: Biol Psychiatry. 2019 May 1;85(9):726–34. doi: 10.1016/j.biopsych.2019.01.017 (PMC6467837; doi:10.1016/j.biopsych.2019.01.017)
Supplement: Supplemental Material [file mmc1.pdf]

A Randomised Trial Directly Comparing Ventral Capsule and Anteromedial Subthalamic Nucleus Stimulation in Obsessive-Compulsive Disorder: Clinical and Imaging Evidence for Dissociable Effects

Supplemental Information

Supplemental Methods and Materials

Pre- and post-surgical 1.5T MRI pre-processing

Pre-implantation MPRAGE scans were brain extracted using *BET* (Brain Extraction Tool, FSL v5.0) which deletes non-brain tissue from a whole head MRI (1). Two-step transformation was used to register native scans to the MNI152 standard-space T1-weighted average structural template image (1mm resolution; (2)). The first step employed linear (affine) transformation using *FLIRT* (FMRIB's Linear Image Registration Tool) with 12 degrees of freedom, correlation ratio cost function and normal search (3, 4). The output from this step was used to execute non-linear registration (second step) using *FNIRT* (FMRIB's Non-Linear Image Registration Tool). This process produced individual native to standard (MNI space) non-linear warp fields which were then applied to the DBS activation volumes acquired from SureTune® to transform all volumes to standard space.

Diffusion weighted 3T MRI acquisition and pre-processing

Diffusion images were acquired using Siemens' 511E Advanced Echo Planar Imaging (EPI) Diffusion WIP. In-plane acceleration was used (GRAPPA factor of 2) with partial Fourier 6/8. In-plane resolution was 1.5×1.5 mm^2 (Field of view 219×219 mm^2, TR = 12200 ms, TE = 99.6 ms) and 85 1.5 mm thick slices were acquired. Diffusion-weighting with b=1500 s/mm^2 was applied along 128 directions uniformly distributed on the sphere and seven b=0s volumes were acquired. To correct for distortions, all acquisitions were repeated with a reversed phase encoding direction (left to right and right to left phase encode) giving a total of 270 volumes acquired ([128 +7] × 2). Total acquisition time was 62 minutes.

The diffusion data were acquired with reversed phase-encode blips (left-to-right and right-to-left), resulting in pairs of images with distortions going in opposite directions. From these pairs the susceptibility-induced off-resonance field was estimated (4, 5). The two images were combined into a single corrected one using *Topup* (FSL v5.0), a tool for estimating

and correcting susceptibility induced distortions prevalent in EPI-DWI. The output from *Topup* was then fed into Eddy (FSL v5.0) for correction of eddy current distortions and subject movement (3).

All diffusion weighted imaging (DWI) scans were imported from DICOM (Digital Imaging and Communications in Medicine) files to NifTI volumes and the diffusion gradient direction values and vectors were extracted using *Volconv* (MJ White, NHNN Neuroradiology Department, London UK).

Patient averaged distortion corrected b=0 volume was registered to brain extracted structural image in native patient space (pre-implantation MPRAGE) with *Flirt* (FSL v5.0) using linear registration with six degrees of freedom, normal search and correlation ratio cost function. The resultant transformation matrices were concatenated with the transformation matrices previously generated using non-linear registration between the structural in native patient space and the standard MNI152-1mm space producing diffusion-to-standard space transformation matrices and their corresponding inversions.

Parametric statistical analysis

Clinical and cognitive measures were checked for homogeneity of variance (Mauchly's test). IED errors were log-transformed because of large baseline individual variability (6). Repeated measures ANOVA was used to test DBS effects during the double-blind cross-over phases comparing baseline, amSTN and VC/VS. Significance levels were adjusted for multiple comparisons using the false discovery rate (FDR) method of Benjamini and Hochberg (7). Post-hoc pair-wise LSD for significant effects were corrected for the FDR.

The effect of time, stimulation at both sites and adjunctive CBT were assessed with repeated measures ANOVA across the six time points. Time points 2 and 3 included amSTN and VC/VS data in the order of randomisation. To test the effect of DBS of amSTN and VC/VS together, phases when one site was stimulated (Time 2+Time 3) was compared to phases when both sites were stimulated (Time 4+Time 5). To test the effect of CBT, Time 5 was compared with Time 6. FDR corrections of significance values were applied throughout.

Effect sizes are given as Cohen's d.

Supplemental Results

Parametric comparison of VC/VS and amSTN DBS

Sphericity was intact for all measures. There were significant improvements in Y-BOCS and MADRS following DBS. All participants completed the IED task and the only significant DBS effect was an improvement in EDS errors. All ANOVAs remained significant controlling for FDR (Y-BOCS: $F(2,10) = 16.59$, $p = 0.003$, $d = 3.66$; MADRS: $F(2,10) = 4.80$; $p = 0.035$, $d = 1.96$; EDS errors: $F(2,10) = 6.01$, $p = 0.029$, $d = 2.19$). Y-BOCS scores significantly improved following both amSTN and VC/VS DBS (Baseline versus amSTN: $p = 0.020$; Baseline versus VC/VS: $p = 0.012$; amSTN versus VC/VS: $p = 0.262$). Changes in MADRS scores were significant for VC/VS but not amSTN DBS (Baseline versus amSTN: $p = 0.277$; Baseline versus VC/VS: $p = 0.051$; amSTN versus VC/VS: $p = 0.051$). Changes in EDS errors were significant for amSTN but not VC/VS DBS (Baseline versus amSTN: $p = 0.039$; Baseline versus VC/VS: $p = 0.741$; amSTN versus VC/VS: $p = 0.054$).

Parametric comparisons of the effects of time, combined DBS, and adjunctive CBT

Sphericity was violated for Y-BOCS and MADRS and Greenhouse-Geisser corrections were applied. Following FDR correction across all three ANOVAs, there was a significant improvement in Y-BOCS and MADRS over time and EDS errors did not change (Y-BOCS: $F(2.21,11.04) = 20.73$, $p=0.003$, $d = 4.08$; MADRS: $F(1.61, 8.05) = 6.16$; $p = 0.042$, $d = 2.21$; EDS errors: $F(5, 25) = 1.57$, $p = 0.217$). Following FDR correction across post-hoc comparisons, there were no statistically significant changes when combined DBS of both sites was compared to single site stimulation (Y-BOCS: $p=0.084$; MADRS: $p=0.154$) or when DBS at both sites was compared to adjunctive CBT (Y-BOCS: $p=0.084$; MADRS $p=0.173$).

Table S1. Active Left (L) and right (R) electrode contacts and amplitudes (V) at the end of each stimulation phase: subthalamic nucleus (amSTN); ventral capsule/ventral striatum (VC/VS), combined amSTN and VC/VS (BOTH); optimal combined settings (OPT); OPT plus adjunctive cognitive behavioural therapy (AdCBT). amSTN was the initial condition for participants 4, 5 and 6. There were 4 electrode contacts on each side, labelled 0, 1, 2, 3, with 0 being the most ventral. Throughout the study the pulse width was 60µs and frequency 130Hz.

**Patient**	**amSTN**	**VC/VS**	**COMB**	**OPT**	**AdCBT**
**1**	L C0-1 1.8V<br>R C1-2 1.8V	L C2-3 7.0V<br>R C2-3 7.0V	**amSTN**<br>L C1 0.7V<br>R C1 0.7V<br>**VC/VS**<br>L C2-3 7.0V<br>R C2-3 7.0V	**amSTN:**<br>L C1 0.7V<br>R C1 0.7V<br>**VC/VS**<br>L C2-3 7.0V<br>R C2-3 7.0V	**amSTN**<br>L C1 0.7V<br>R C1 0.7V<br>**VC/VS**<br>L C2-3 7.0V<br>R C2-3 7.0V
**2**	L C0 2.35V<br>R C0 2.4V	L C2-3 6.5V<br>R C2-3 6.5V	**amSTN**<br>L C0 2.45V<br>R C0 2.45V<br>**VCVS**<br>L C2-3 4.1V<br>R C2-3 4.1V	**amSTN**<br>L C0 2.85V<br>R C0 2.85V<br>**VC/VS**<br>L C2-3 4.0V<br>R C2-3 4.0V	**amSTN**<br>L C0 2.85V<br>R C0 2.85V<br>**VC/VS**<br>L C2-3 4.0V<br>R C2-3 4.0V
**3**	L C0 2.6V<br>R C0 2.6V	L C2-3 6.5V<br>R C2-3 6.5V	**amSTN**<br>L C0 2.3V<br>R C0 2.3V<br>**VC/VS**<br>L C2-3 5.7V<br>R C2-3 5.7V	**amSTN**<br>L C0 2.3V<br>R C0 2.3V<br>**VC/VS**<br>L C2-3 5.4V<br>R C2-3 5.4V	**amSTN**<br>L C0 2.3V<br>R C0 2.3V<br>**VC/VS**<br>L C2-3 5.4V<br>R C2-3 5.4V
**4**	L C0-1 1.5V<br>R C0-1 1.5V	L C2-3 6.2V<br>R C2-3 6.2V	**amSTN:**<br>L C0-1 1.3V<br>R C0-1 1.3V<br>**VC/VS:**<br>L C2-3 6.2V<br>R C2-3 6.2V	**amSTN:**<br>L C0-1 1.3V<br>R C0-1 1.3V<br>**VC/VS:**<br>L C2-3 6.4V<br>R C2-3 6.4V	**amSTN**<br>L C0-1 1.3V<br>R C0-1 1.3V<br>**VC/VS**<br>L C2-3 6.4V<br>R C2-3 6.4V
**5**	L C0 1.4V<br>R C0 1.4V	L C2-3 7.0V<br>R C2-3 7.0V	**amSTN**<br>L C0 1.25V<br>R C0 1.25V<br>**VC/VS**<br>L C2-3 7.0V<br>R C2-3 7.0V	**amSTN**<br>L C2 1.25V<br>R C0 1.25V<br>**VC/VC**<br>L C2-3 7.0V<br>R C2-3 7.0V	**amSTN**<br>L C2 1.25V<br>R C0 1.25V<br>**VC/VC**<br>L C2-3 7.0V<br>R C2-3 7.0V
**6**	L C0 1.5V<br>R C1 1.5V	L C2 5.5V<br>R C2 5.4V	**amSTN**<br>L C0 0.8V<br>R C1 0.8V<br>**VC/VS**<br>L C2 4.5V<br>R C2 4.5V	**amSTN**<br>L C0 1.0V<br>R C1 1.0V<br>**VC/VS**<br>L C2 5.3V<br>R C2 5.3V	**amSTN**<br>L C0 1.0V<br>R C1 1.0V<br>**VC/VS**<br>L C2 5.3V<br>R C2 5.3V

Table S2. Average volume of tissue activation (VTA) and coordinates of maximum intensity and centre of gravity voxel in MNI-152 space.

	Maximum intensity (vox)			Center of gravity (vox)			Volume (mm^3)
	X	Y	Z	X	Y	Z	
Left VC	102	135	67	103	134	70.8	831
Right VC	77	134	66	77.4	134	69.9	716
Left amSTN	97	112	63	97.9	112	62.6	117
Right amSTN	81	113	64	81.6	113	62.9	128

VC: ventral capsule; amSTN: anteromedial subthalamic nucleus; vox: voxel; MNI-152: Montreal Neurological Institute 152 average brain ($1mm^3$).

Table S3. Individual patient adverse events during amSTN, VC/VS and combined DBS phases. 'Combined' includes three phases: COMB, OPT, AdCBT. The events are listed as to whether they were considered DBS related or unrelated.

**Postoperative period**	**amSTN**		**VC/VS**		**Combined amSTN+VC/VS**	
	**Stimulation related**	**Stimulation unrelated**	**Stimulation related**	**Stimulation unrelated**	**Stimulation related**	**Stimulation unrelated**
Scalp wound site hypoaesthesia<br><br>Urinary retention	Mild hypomania<br><br>Low mood<br><br>Restlessness<br><br>Hypomania<br><br>Hypersexual thoughts<br><br>Insomnia<br><br>Severe anxiety requiring reverting to VC/VS after 2 weeks<br><br>Readmission for readjustment – worsening OCD and mood	Insomnia<br><br>Unauthorised medication change resulting in nausea and diarrhoea<br><br>Fall and wrist fracture	Hypomania with alcohol excess and minor hand fracture<br><br>Mild hypomania<br><br>Irritability and poor sleep<br><br>Readmission for readjustment – worsening OCD and mood	Unauthorised medication change<br><br>Restless legs	Hypomania (COMB)<br><br>Hypomania (COMB)<br><br>Mild hypomania (COMB)<br><br>Restlessness (COMB)<br><br>Readmission – hypomanic symptoms reported (OPT)<br><br>Readmission – worsening OCD (OPT)<br><br>Hypersexual thoughts (OPT)<br><br>Facial tic (OPT)	Restless legs treatment (COMB)<br><br>Readmission - unwell after knee surgery, post-op infection and unauthorised medication change (OPT)<br><br>Dry eyes (CBT)

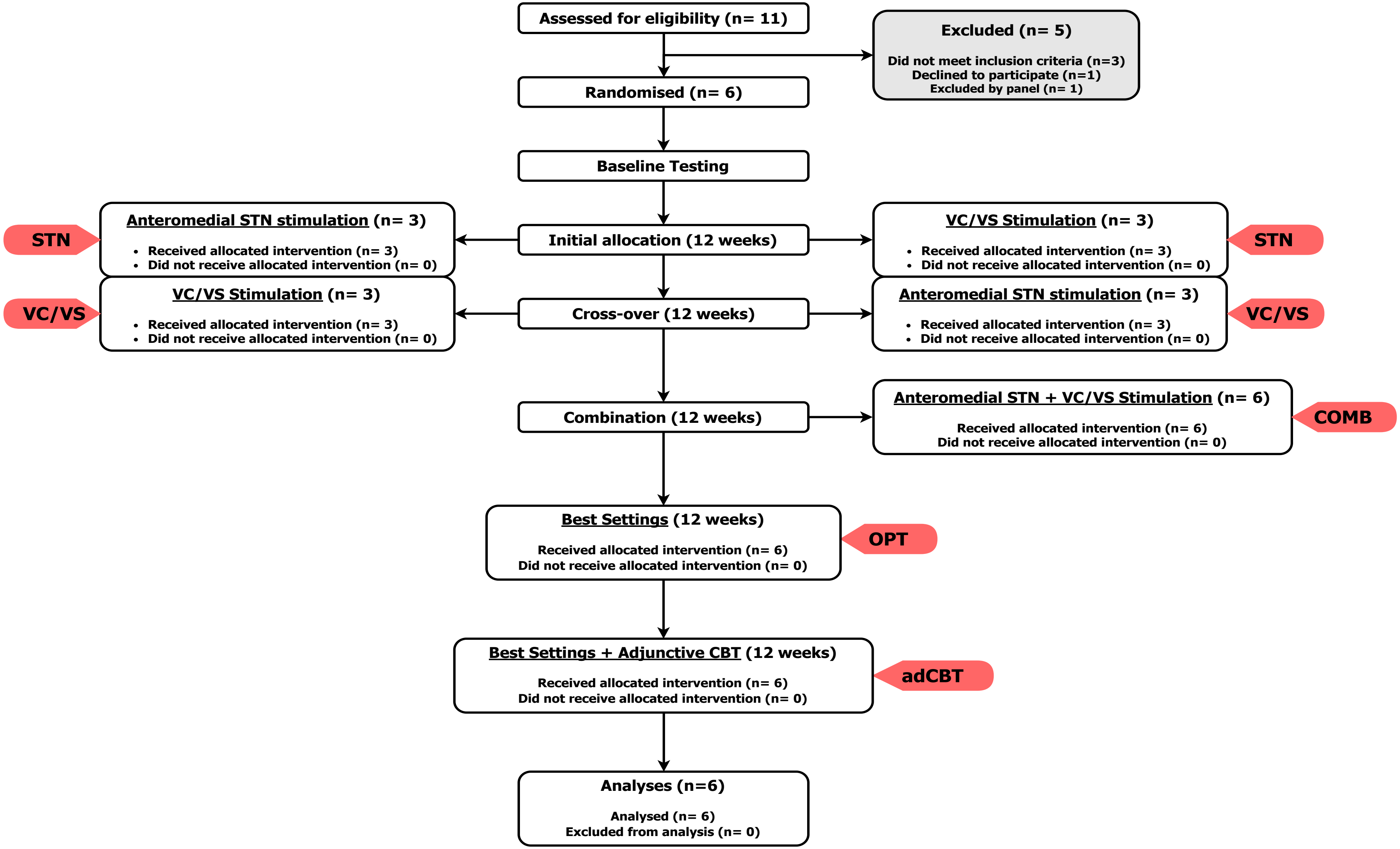

Figure S1. CONSORT diagram: Trial structure and patient flow

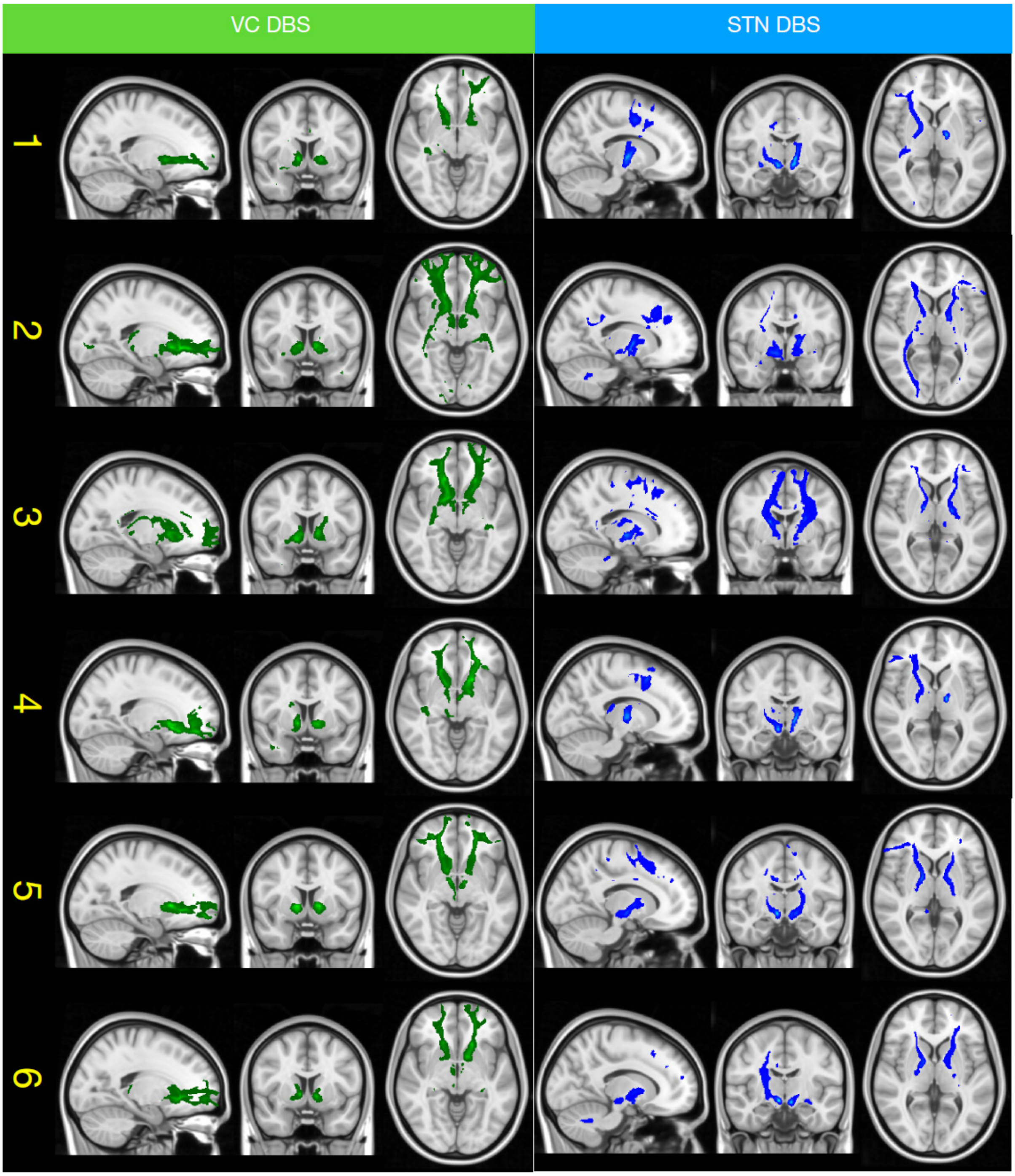

Figure S2. Tractography from the amSTN-VTA and VC-VTA of individual patients shown in MNI space.

Supplemental References

1 Smith SM. (2002): Fast robust automated brain extraction. *Hum Brain Mapp*. 17(3): 143–155.

2. Grabner G, Janke AL, Budge MM, Smith D, Pruessner J, Collins DL. (2006): Symmetric atlasing and model based segmentation: an application to the hippocampus in older adults. *Med Image Comput Comput Assist Interv*.9(Pt 2):58-66.
3. Andersson JLR, Sotiropoulos SN. (2016): An integrated approach to correction for off-resonance effects and subject movement in diffusion MR imaging. *Neuroimage*.125:1063-1078.
4. Smith SM, Jenkinson M, Woolrich MW, Beckmann CF, Behrens TE, Johansen-Berg H*, et al.* (2004): Advances in functional and structural MR image analysis and implementation as FSL. *Neuroimage*.23 Suppl 1:S208-219.
5. Andersson JL, Skare S, Ashburner J. (2003): How to correct susceptibility distortions in spin-echo echo-planar images: application to diffusion tensor imaging. *Neuroimage*.20(2):870-888.
6. van den Berg RA, Hoefsloot HC, Westerhuis JA, Smilde AK, van der Werf MJ. (2006): Centering, scaling, and transformations: improving the biological information content of metabolomics data. *BMC Genomics*.7:142.
7. Benjamini Y, Hochberg Y. (1995): Controlling the false discovery rate: A practical and powerful approach to multiple testing. *Journal of the Royal Statistical Society Series B (Methodological)*.57(1):289-300.
